# Proteomic analysis of HDL from inbred mouse strains implicates APOE associated with HDL in reduced cholesterol efflux capacity via the ABCA1 pathway[S]

**DOI:** 10.1194/jlr.M063701

**Published:** 2016-02

**Authors:** Nathalie Pamir, Patrick Hutchins, Graziella Ronsein, Tomas Vaisar, Catherine A. Reardon, Godfrey S. Getz, Aldons J. Lusis, Jay W. Heinecke

**Affiliations:** Department of Medicine,* University of Washington, Seattle, WA; Department of Pathology,† University of Chicago, Chicago, IL; Department of Genetics,§ University of California at Los Angeles, Los Angeles, CA

**Keywords:** atherosclerosis, cardiovascular risk, mass spectrometry, high density lipoprotein, apolipoprotein E, ATP binding cassette transporter A1

## Abstract

Cholesterol efflux capacity associates strongly and negatively with the incidence and prevalence of human CVD. We investigated the relationships of HDL’s size and protein cargo with its cholesterol efflux capacity using APOB-depleted serum and HDLs isolated from five inbred mouse strains with different susceptibilities to atherosclerosis. Like humans, mouse HDL carried >70 proteins linked to lipid metabolism, the acute-phase response, proteinase inhibition, and the immune system. HDL’s content of specific proteins strongly correlated with its size and cholesterol efflux capacity, suggesting that its protein cargo regulates its function. Cholesterol efflux capacity with macrophages strongly and positively correlated with retinol binding protein 4 (RBP4) and PLTP, but not APOA1. In contrast, ABCA1-specific cholesterol efflux correlated strongly with HDL’s content of APOA1, APOC3, and APOD, but not RBP4 and PLTP. Unexpectedly, APOE had a strong negative correlation with ABCA1-specific cholesterol efflux capacity. Moreover, the ABCA1-specific cholesterol efflux capacity of HDL isolated from APOE-deficient mice was significantly greater than that of HDL from wild-type mice. Our observations demonstrate that the HDL-associated APOE regulates HDL’s ABCA1-specific cholesterol efflux capacity. These findings may be clinically relevant because HDL’s APOE content associates with CVD risk and ABCA1 deficiency promotes unregulated cholesterol accumulation in human macrophages.

Clinical and epidemiological studies show a robust inverse association of HDL-cholesterol (HDL-C) levels with CVD risk (1). In randomized clinical trials, however, two drugs, CETP inhibitors and niacin, that elevate HDL-C levels by different mechanisms, failed to reduce CVD risk in statin-treated humans with established atherosclerosis (2, 3). These observations indicate that raising HDL-C levels does not necessarily improve HDL’s cardioprotective functions. It is therefore critical to develop new metrics for assessing HDL’s functionality in humans (2).

Many lines of evidence support the proposal that mediation of cholesterol efflux from macrophages is one of HDL’s important functions (4). This process is the first step of reverse cholesterol transport, in which HDL accepts cholesterol from cells and carries it back to the liver for excretion. Two cellular pathways for cholesterol efflux involve the membrane ABC transporters, ABCA1 and ABCG1, which are highly induced when macrophages accumulate excess cholesterol (5, 6). ABCA1 exports phospholipids and cholesterol from the cell membrane to lipid-poor apolipoproteins (6) and to small dense HDL (7), while ABCG1 exports cholesterol to mature HDL particles (5, 6). Humans with Tangier disease, who are deficient in ABCA1, accumulate cholesterol-loaded macrophages in many different tissues (8, 9). Moreover, mouse studies demonstrate that defects in the apolipoprotein/ABCA1 pathway are important determinants of cholesterol accumulation by macrophages (5).

It has been difficult to assess the clinical relevance of cholesterol efflux. However, recent studies of cAMP-stimulated J774 macrophages radiolabeled with cholesterol, which measures cellular efflux by multiple pathways (ABCA1, ABCG1, SR-B1, and diffusion) (10), demonstrate strong inverse correlations between cholesterol efflux capacity of serum HDL (serum depleted of APOB-containing lipoproteins) and prevalent coronary artery disease (11). Moreover, macrophage efflux capacity remained a strong predictor of prevalent coronary artery disease after adjustment for HDL-C levels (11).

The cholesterol efflux capacity of serum HDL with cAMP-stimulated J774 macrophages can also be assessed with fluorescently labeled cholesterol, which primarily measures ABCA1-mediated efflux from these cells (12). A recent study of a population-based cohort that was free of CVD demonstrated that ABCA1-specific cholesterol efflux capacity assessed by this method associates strongly and negatively with the risk of future cardiac events (13). This association persisted after multivariate adjustment, suggesting that altered HDL function affects cardiovascular risk by processes distinct from those involving HDL-C or traditional lipid risk factors. Impaired cholesterol efflux capacity with J774 macrophages radiolabeled with cholesterol also predicted incident cardiac events in a healthy cohort (14). Taken together, these observations provide strong evidence that cholesterol efflux capacity is a better measure of HDL’s cardioprotective effects than HDL-C.

The molecular factors that control HDL’s serum efflux capacity are poorly understood (10, 12). For example, HDL-C predicts only 34% of serum HDL’s macrophage cholesterol efflux capacity (11); HDL-C fails to predict HDL’s ABCA1-specific cholesterol efflux capacity with cells labeled with fluorescent cholesterol (12, 13). Macrophage cholesterol efflux capacity of serum HDL also correlates poorly with plasma APOA1 levels (11), even though APOA1 is HDL’s major structural protein, accounting for ∼70% of protein mass. In human HDL, APOA2 accounts for ∼20% of HDL’s protein, while >80 proteins account for the other 10% of HDL’s proteome, as assessed by MS (15, 16). Moreover, various HDL subspecies have very different proteomes (17, 18). It is therefore unclear which, if any, of the proteins contribute to HDL’s proposed cardioprotective effects.

In the current studies, we used MS and HDL isolated by ultracentrifugation from five inbred mouse strains that differed in HDL-C levels and atherosclerotic susceptibility (19) to investigate the relationships among HDL’s size, protein composition, and cholesterol efflux capacity, using both J774 macrophages and BHK cells expressing ABCA1. Our unbiased approaches identified candidate proteins that correlate with HDL’s size and with its cholesterol efflux capacity with macrophages and the ABCA1 pathway. Importantly, we used mice deficient in APOE and APOA2 to confirm that these proteins influence HDL’s efflux capacity and size.

## METHODS

### Mice

All studies were approved by the Animal Care and Use Committees of the University of Washington and the University of California, Los Angeles. Mice were housed (three to five per cage) in a specific pathogen-free barrier facility (22°C) with a 12 h light/dark cycle with free access to food and water. All the strains were a fed low-fat diet (Wayne Rodent BLOX 8604; Harlan Teklad Laboratory) until they were 8 weeks old. Then mice that were fasted for 4 h in the morning were bled from the retro-orbital sinus into tubes containing EDTA (final concentration 1 mM) and euthanized by isofluorane inhalation. Plasma was collected and stored at −80°C until analysis.

### Plasma lipid and APOA1 measurements

Triglycerides and phospholipids (Wako Diagnostics) and cholesterol levels (Invitrogen) were determined biochemically. APOA1 levels were determined by immunoblot analysis with a goat anti-mouse APOA1 antibody (US Biological).

### Cholesterol efflux assays

Macrophage cholesterol efflux capacity was assessed with J774 macrophages labeled with [^3^H]cholesterol and stimulated with a cAMP analog, as described by Rothblat and colleagues (10). Efflux via the ABCA1 or ABCG1 pathways was measured with BHK cells expressing mifepristone-inducible human ABCA1 or ABCG1 that were radiolabeled with [^3^H]cholesterol (20). Efflux of [^3^H]cholesterol was measured after a 4 h incubation in medium with APOB-depleted serum HDL (2.8% v/v) or isolated HDL (30 μg protein per milliliter). ABCA1-specific cholesterol efflux capacity was calculated as the percentage of total [^3^H]cholesterol (medium plus cell) released into the medium of BHK cells stimulated with mifepristone after the value obtained with cells stimulated with medium alone was subtracted.

### HDL isolation

Serum HDL was prepared by adding calcium (2 mM final concentration) to plasma and using polyethylene glycol (8 kDa; Sigma) to precipitate lipoproteins containing APOB (VLDL, IDL, LDL). After centrifugation at 10,000 *g* for 30 min at 4°C, serum HDL was harvested from the supernatant. HDL was isolated from serum or EDTA-anticoagulated plasma using sequential ultracentrifugation (d = 1.063–1.21 mg/ml) (15, 21). HDL was stored on ice in the dark and used within 1 week of preparation.

### LC-ESI-MS/MS analysis

HDL (10 μg protein) isolated by ultracentrifugation was solubilized with 0.1% RapiGest (Waters) in 200 mM ammonium bicarbonate, reduced with dithiothreitol, alkylated with iodoacetamide, and digested with trypsin (1:20, w/w HDL protein; Promega) for 3 h at 37°C. After a second aliquot of trypsin (1:20, w/w HDL protein) was added, samples were incubated overnight at 37°C. After RapiGest was removed by acid hydrolysis, samples were dried and stored at −20°C until analysis. Prior to analysis, samples were reconstituted in 5% acetonitrile and 0.1% formic acid (15, 18).

Tryptic digests of mouse HDL (1 μg protein) were injected onto a C18 trap column (Paradigm Platinum Peptide Nanotrap, 0.15 × 50 mm; Michrom BioResources, Inc., Auburn, CA), desalted (50 μl/min) for 5 min with 1% acetonitrile/0.1% formic acid, eluted onto an analytical reverse-phase column (0.15 × 150 mm, Magic C18AQ, 5 μm, 200 Å Michrom BioResources, Inc.), and separated on a Paradigm M4B HPLC (Michrom BioResources, Inc.) at a flow rate of 1 μl/min over 180 min, using a linear gradient of 5–35% buffer B (90% acetonitrile, 0.1% formic acid) in buffer A (5% acetonitrile, 0.1% formic acid). ESI was performed using a CaptiveSpray source (Michrom BioResources, Inc.) at 10 ml/min flow rate and 1.4 kV setting. HDL digests were introduced into the gas phase by ESI, positive ion mass spectra were acquired with a linear ion trap mass spectrometer (LTQ; Thermo Electron Corp.) using data-dependent acquisition (one MS survey scan followed by MS/MS scans of the eight most abundant ions in the survey scan) with a *m/z* 400–2,000 scan. An exclusion window of 45 s was used after two acquisitions of the same precursor ion (15, 18).

### Protein identification

MS/MS spectra were matched against the mouse International Protein Index database (mouse v.3.54), using the SEQUEST (version 2.7) search engine with fixed Cys carbamidomethylation and variable Met oxidation modifications. The mass tolerance for precursor ions was 2.5 ppm; SEQUEST default tolerance was 2.5 Da for precursor ion mass and 1 Da for fragment ion mass. SEQUEST results were further validated with PeptideProphet and ProteinProphet (22, 23), using an adjusted probability of ≥0.90 for peptides and ≥0.95 for proteins. Each charge state of a peptide was considered a unique identification.

We used the gene and protein names in the Entrez databases [National Center for Biotechnology Information; based on the nomenclature guidelines of the Human Gene Nomenclature Committee (http://www.gene.ucl.ca.uk/nomenclature) for human guidelines (24), and Mouse Genome Informatics (http://www.infromatics.jax.org.nomen/) for mouse guidelines (25)] to identify HDL proteins and to eliminate the redundant identifications of isoforms and protein fragments frequently found in databases used in proteomic analysis (26).This approach also permits cross-referencing of proteins from different species. When MS/MS spectra could not differentiate between protein isoforms, the isoform with the most unique peptides was used for further analysis.

### Protein quantification

Proteins were quantified using spectral counts, the total number of MS/MS spectra detected for a protein (15, 27, 28). Proteins considered for analysis had to be detected in three or more analyses with two or more unique peptides. When MS/MS spectra could not differentiate between protein isoforms, the isoform with the most unique peptides was used for further analysis. Spectral counts for each protein, normalized to total spectral counts for peptides from each sample, were used to calculate a spectral index to compare the relative protein composition of mouse strains’ HDLs (15). We included in our analyses only proteins that were detected in 75% of samples in at least one mouse strain. Supplementary Table 1 provides the total calculated spectral counts for each protein, the individual peptides that identified each protein, the total number peptide spectra counts, and relative quantification.

### HDL particle concentration and size

HDL particle concentration and size (HDL-P_ima_) were quantified by calibrated ion mobility analysis (IMA) (29). Briefly, HDL isolated by ultracentrifugation from EDTA plasma was introduced into the gas-phase ions by ESI. The resulting highly charged ions were largely neutralized by α particles, yielding a small proportion of singly charged cations, which were introduced into the mobility analyzer. As the particles moved through a strong electromagnetic field, they were separated according to their electrophoretic mobility and then enumerated by a particle counter. HDL peak areas were converted into aqueous particle concentrations using glucose oxidase calibration curves.

Because electrophoretic mobility depends chiefly on size, IMA data are expressed in terms of particle diameter (nanometers), which corresponds to the calculated diameter of a singly charged spherical particle with the same electrophoretic mobility (29). The method yields a stoichiometry of APOA1 and the sizes and relative abundance of HDL subspecies in excellent agreement with those determined by nondenaturing gradient gel electrophoresis and analytical ultracentrifugation (29, 30). Because calibrated IMA accurately quantifies the size and concentration of gold nanoparticles and reconstituted HDL (29), we term the concentration and size of HDL determined by this method, HDL-P_ima_.

### Statistical analyses

Data are represented as mean ± SEM. Differences among the HDLs of the five groups were assessed with ANOVA followed by Fisher’s exact test to correct for multiple comparisons. The results of ANOVA are presented as F(df_1_, df_2_), where F is the test statistic of the F distribution, and df_1_ and df_2_ are the degrees of freedom of the analysis. The Kruskal-Wallis one-way ANOVA by ranks followed by Dunn’s test (to correct for multiple comparisons) was used for proteomic analyses. Linear correlations were assessed with Pearson’s product-moment coefficient. *P* < 0.05 was considered significant. Data were analyzed with Prism and R software.

## RESULTS

### HDL’s protein cargo is genetically controlled

Structural and quantitative variations in APOA1 and APOA2 of HDL isolated from inbred strains of mice have been previously reported (31, 32). To test the hypothesis that the HDLs in different strains of mice have different protein cargos that affect size and function, we used shotgun proteomics to analyze HDL isolated from the plasma of five inbred mouse strains that differed in atherosclerosis susceptibility (least to most susceptible: NZW/LacJ < SWR/J < DBA/2J < C57BL/6J < C57BLKS/J). The HDLs were isolated by ultracentrifugation and digested with trypsin, and the peptide digest was analyzed by LC-ESI-MS/MS. Judged by stringent statistical criteria (Methods), shotgun proteomics identified 72 proteins with high confidence in one or more mouse strain (supplementary Table 1).

**Fig. 1.**
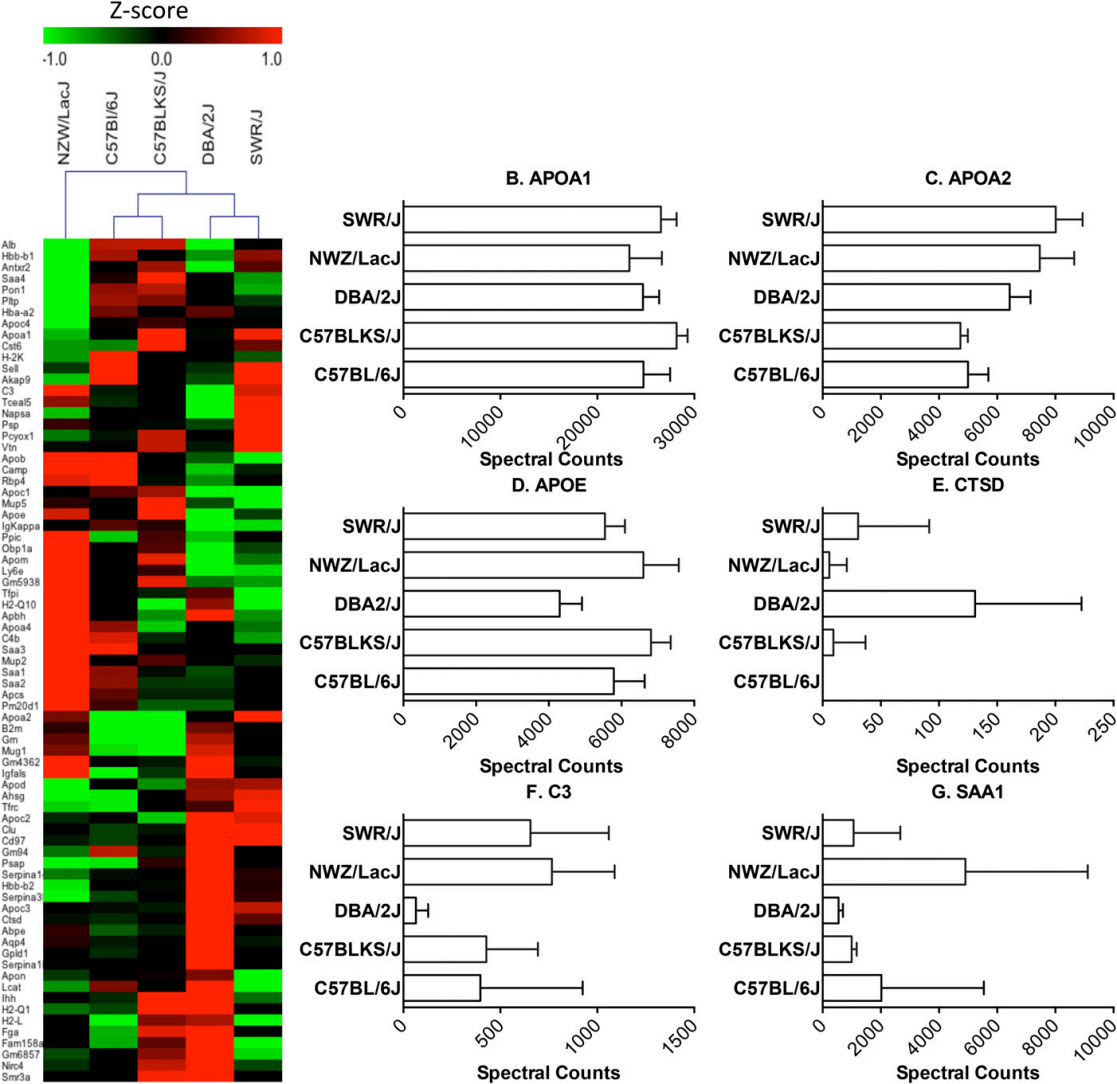
The HDL proteome of inbred mouse strains. LC-ESI-MS/MS analysis of proteins in HDL isolated by ultracentrifugation from five different strains of mice. Proteins were quantified by spectral counting (total number of peptides identified for a given protein normalized to total spectral counts). A: Heat map of differentially expressed proteins. Relative protein abundance was calculated as z-scores that were generated from adjusted spectral counts. Red, upregulated; green, downregulated. Mouse strains were clustered by Euclidian hierarchical cluster analysis. B–G: Quantification of representative mouse HDL proteins.

Hierarchical clustering, as assessed by Euclidean distance analysis, of the HDL proteome recapitulated the genealogy of the strains, as determined by high density single nucleotide polymorphism genotyping (33).Thus, C57BL/6J and C57BLKS/J are most closely related to each other and these are then most related to DBA/2J, SWR/J, and NZW/LacJ, in that order (**Fig. 1A**). These observations support the proposal that a wide range of proteins in HDL are under genetic control.

Sixty of the 72 proteins were detected in all five strains, and 45 of the 60 were differentially expressed (supplementary Table 1, Kruskal-Wallis, *P* < 0.05; e.g., APOA2, APOE, APOC3, APOD, SAA1, SAA2, C3, PLTP). Adjusted spectral counts of representative proteins for five strains are presented in Fig. 1B–G. DBA/2J had the most diverse proteome (Fig. 1A). For example, GPLD1, AQP4, SERPINA1b, and CTSD were expressed at high levels in HDL from the DBA/2J mice, but not in the other strains of mice, while three proteins that were expressed by all the other strains were undetectable in the HDL of DBA/2J mice [retinol binding protein 4 (RBP4), TCEAL5, IGKAPPA].

Cluster analysis suggested that certain proteins are coexpressed on HDL (supplementary Fig. 1), perhaps indicating coordinate regulation or functional complexes. For example, several closely related proteins clustered together. Examples include SAA1 and SAA2 acute-phase response proteins that share a high degree of sequence identity. In contrast, SAA4, which is constitutively expressed, did not cluster with the other SAA isoforms. Histocompatibility 2 Q region locus 10 (H2-Q10), B2-M, HBA-A2, and HBB-B1 also clustered together. These proteins are not members of a single gene family, but each is implicated in immune regulation, raising the possibility that their expression levels in HDL might be coordinately regulated as part of the immune response.

### Mouse HDL proteins are involved in lipid metabolism, immunity, and inhibition of proteolysis

**Fig. 2.**
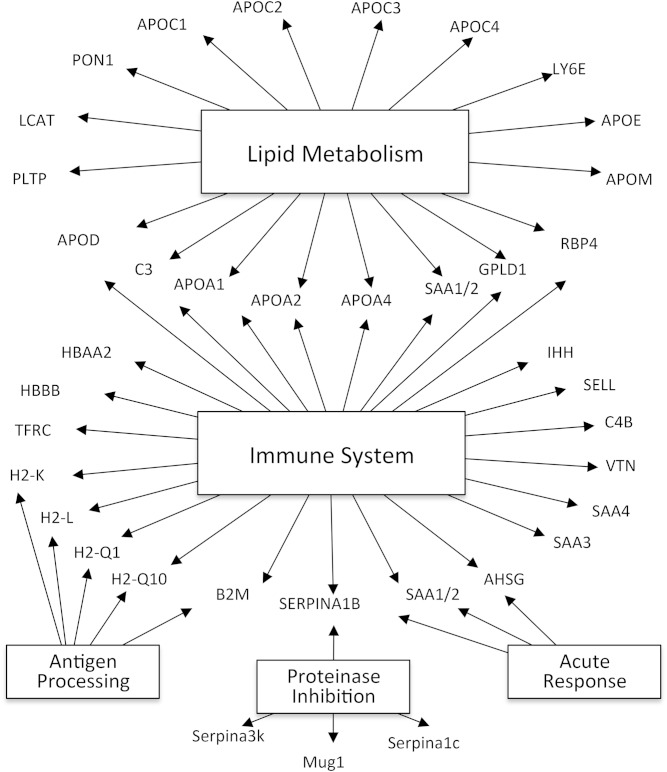
Global view of biological processes and molecular functions of HDL proteins. Proteins detected in HDL were associated with biological functions using Gene Ontology process annotations. This approach demonstrated significant overrepresentation of proteins involved in several categories, including lipid metabolism (*P* = 10^−15^), the immune response (*P* = 10^−6^), protease inhibitor activity (*P* = 10^−7^), antigen processing (*P* = 10^−3^), and the acute-phase response (*P* = 10^−5^).

Gene Ontology analysis (**Fig. 2**) demonstrated that mouse HDL is enriched in proteins associated with lipid metabolism (18 proteins, *P* = 10^−15^), the immune response (25 proteins, *P* = 10^−6^), the acute-phase response (3 proteins, *P* = 10^−5^), and antigen processing (5 proteins, *P* = 10^−3^). Four proteins associated with proteinase inhibition (*P* = 10^−7^). Importantly, the existing annotation databases used for Gene Ontology analysis are incomplete, and only a subset of genes is functionally annotated. Indeed, we have used MS/MS analysis to demonstrate that over half the proteins detected in HDL are acute-phase response proteins in mice injected with silver nitrite (34), a widely used model of inflammation. Collectively, these observations indicate that mouse HDL, like human HDL (15), carries proteins that participate in lipid metabolism, acute inflammation, immunity, and inhibition of proteolysis.

Like human HDL (15, 35), mouse HDL contains major histocompatibility complex proteins. However, mouse HDL appeared to be more enriched in proteins linked to the adaptive immune system, including β-2-microglobulin (the β-chain of the heterodimer that presents major histocompatibility complex class I molecules), H2-Q10, and H2-L, which the human genome lacks. In contrast, we did not detect orthologs of certain human HDL proteins linked to the acute response, such as HPX, ORM2, and TTR, in mouse HDL.

More than 80 proteins have been identified with high confidence in human HDL by at least two independent laboratories (15, 16, 36), However, only 42 of the 72 proteins we detected in mouse HDL have been identified in human HDL. Many of those mouse proteins lack human orthologs. Examples include H2-Q10, APON, H2-L, SERPINA-3K, PSAP, AHSG, and NAPSA. However, Gene Ontology analysis (Fig. 2) revealed that both human (15) and mouse HDLs contain the same major functional categories of proteins. These data demonstrate that the HDL proteomes of mice and humans share similar functions and have many of the same proteins, but are also distinct.

### The size of HDL varies significantly in different strains of mice

**Fig. 3.**
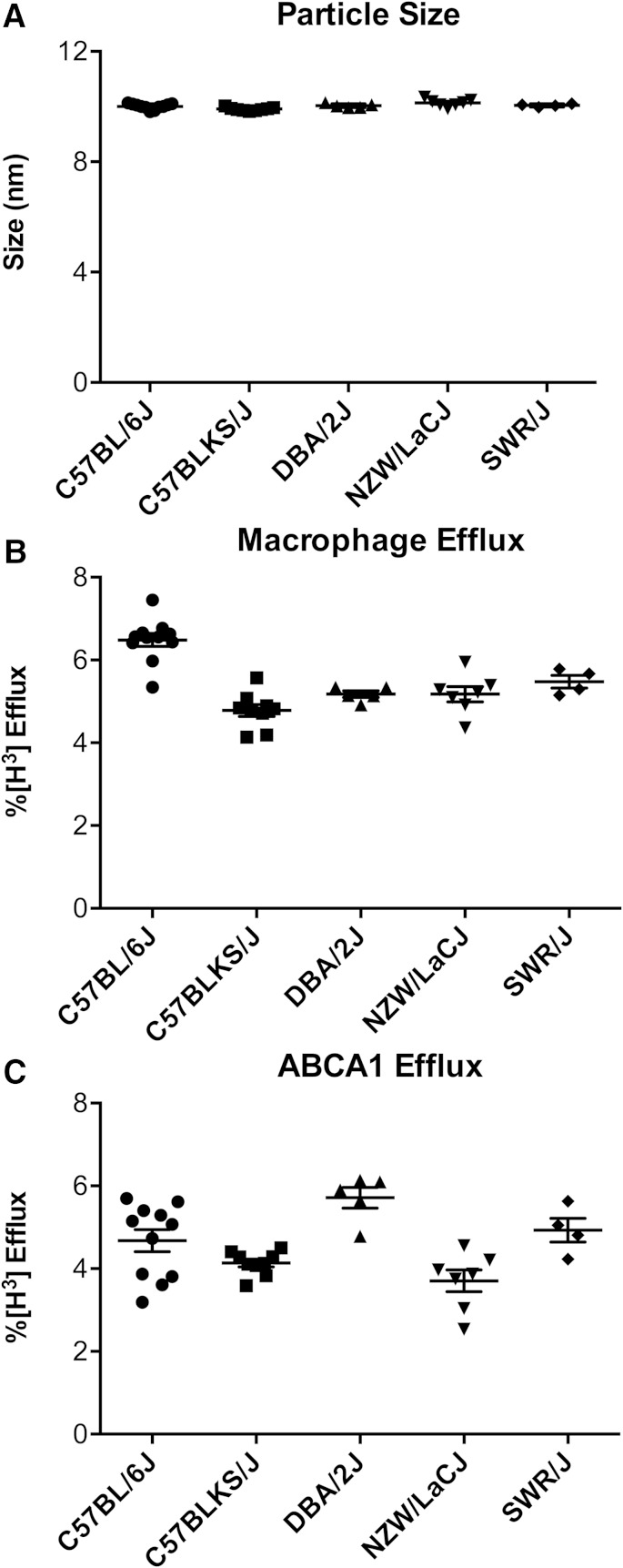
Macrophage cholesterol efflux capacity, ABCA1 efflux capacity, and HDL size in the different strains of mice. A: HDL size was measured by calibrated IMA in HDL isolated from serum by ultracentrifugation. Serum HDL was obtained by polyethylene glycol precipitation of APOB-containing lipoproteins from plasma-derived serum. Macrophage cholesterol efflux capacity (cAMP-stimulated J774 macrophages) (B) and ABCA1-specific cholesterol efflux (mifepristone-stimulated BHK cells minus BHK cells incubated in medium alone) (C) were measured after a 4 h incubation with serum HDL (2.8% v/v) as described in the Methods.

HDL particles in the different strains of mice differed significantly in size [**Fig. 3A**; ANOVA, F(4, 31) = 6.1, *P* < 0.001, and F(4, 31) = 6.2, *P* < 0.001 for size and concentration, respectively]. HDL particles isolated from the NZW/LacJ mice had the largest diameter (10.14 ± 0.13 nm), while HDL from the C57BLKS/J mice had the smallest (9.9 ± 0.05 nm, *P* < 0.0001). Based on calibration with proteins of known molecular mass (29), we estimate the difference in molecular mass between the largest and smallest particles to be ∼10 kDa. It is likely that alterations in HDL particle size reflect changes in particle lipid and/or protein composition. The five strains of mice had significantly different levels of HDL-C, APOA1, and phospholipids in serum HDL (APOB depleted serum; supplementary Fig. 2B–E).

To determine whether any strain-specific compositional differences were present, we measured the HDL phospholipid, free cholesterol, cholesteryl ester, and protein content of particles isolated by ultracentrifugation. We normalized the values to HDL particle concentration (HDL-P_ima_) to estimate composition of HDL particles for each strain (supplementary Fig. 2F). We observed less than 5% differences among proteins, cholesteryl esters, and phospholipids. Free cholesterol varied ∼40% among strains (6.4% vs. 10.1% between DBA2/J and NZW/LacJ, respectively). The lack of major differences in HDL’s core lipids and phospholipids between strains suggests that size differences are due to HDL’s protein composition.

### Candidate HDL proteins that affect HDL size

**Fig. 4.**
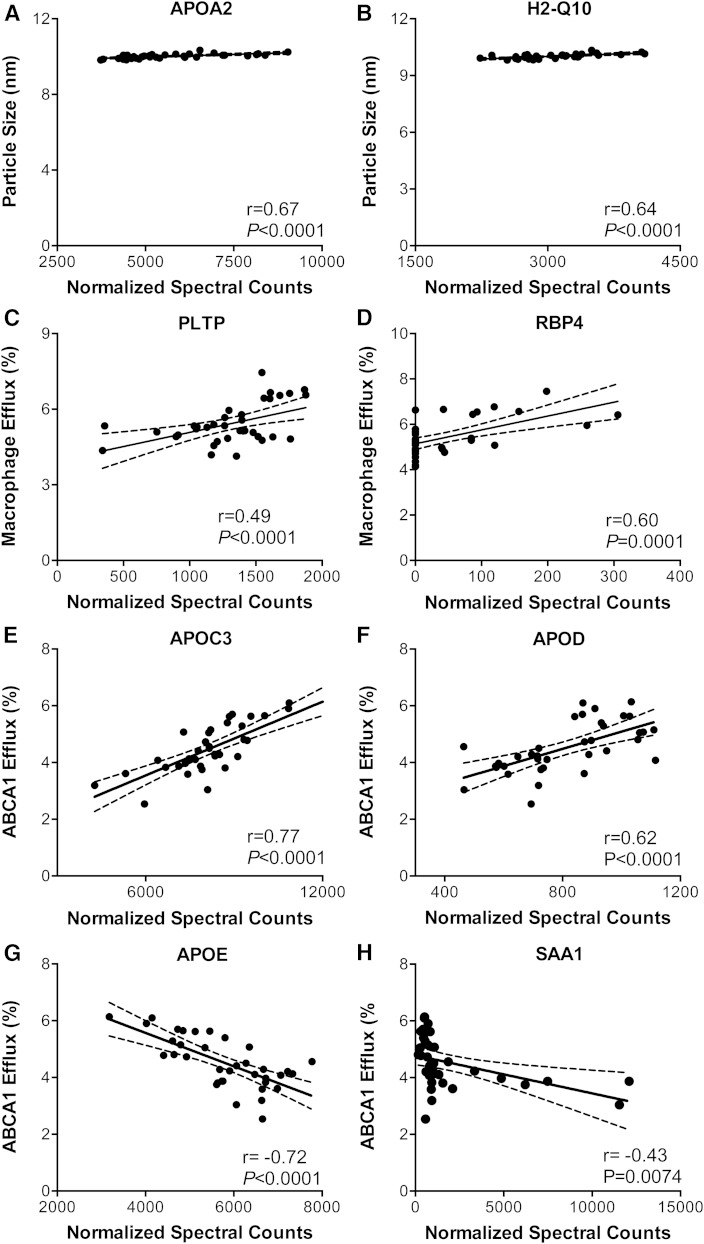
Correlations of HDL protein levels with HDL size, macrophage efflux capacity, and ABCA1 efflux capacity. A, B: HDL size was measured by calibrated IMA in HDL isolated from serum by ultracentrifugation. Macrophage cholesterol efflux capacity of serum HDL (C, D) or ABCA1-specific cholesterol efflux capacity (E–H) were measured with serum HDL as described in the legend to Fig. 3. The relationship of relative HDL protein abundance to HDL size and efflux capacity was quantified by Pearson’s correlation. All proteins with *r* ≥ |0.5| and *P* < 0.001 were included in this analysis.

We next determined whether differential expression of HDL proteins in the five strains of mice correlated with HDL particle size. All five strains of mice had both small (∼10.1 nm) and large (∼12.6 nm) HDLs; the small HDL subspecies was ∼10-fold more abundant than the large subspecies. Particle size correlated strongly with HDL’s content of APOA2 and H2-Q10 (**Fig. 4A, B**; *r* = 0.67 and 0.64, both *P* < 0.0001), but not with that of APOA1 or HDL-C (data not shown). APOA2 is HDL’s second most abundant protein, and H2-Q10 is a component of the murine histocompatibility complex that participates in antigen processing.

### APOA2 regulates HDL particle size

**Fig. 5.**
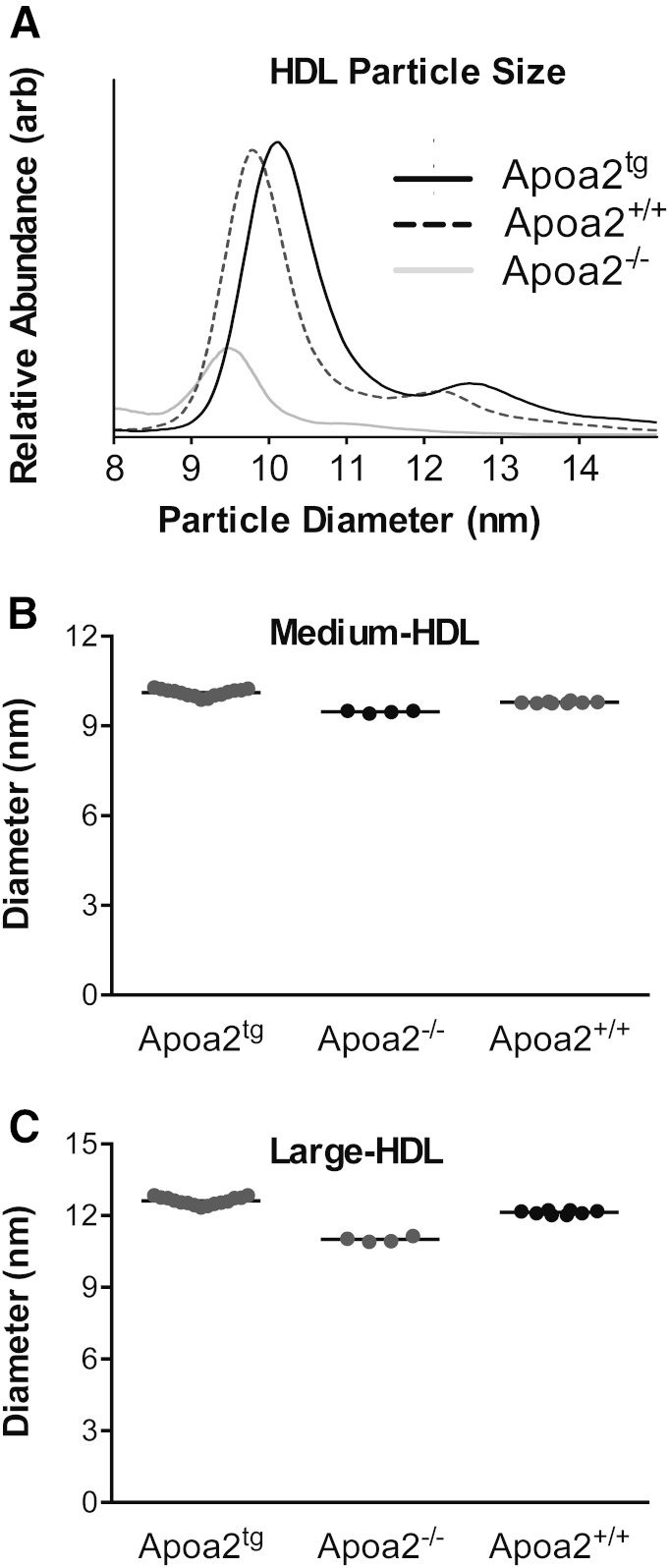
HDL particle size and abundance in wild-type mice, *Apoa2*^−/−^ mice, and human APOA2 transgenic mice. Relative HDL particle abundance (A) and size (B, C) were determined by calibrated IMA in HDL isolated by ultracentrifugation of the plasma of *Apoa2*^tg^ mice (n = 15), *Apoa2^+/+^* mice (n = 8), and *Apoa2*^−/−^ mice (n = 4). arb, arbitrary units.

To determine whether APOA2 controls the size of HDL particles, we isolated HDL by ultracentrifugation of plasma from wild-type mice, *Apoa2*^−/−^ mice, and human *APOA2* transgenic mice (all in the C57BL/6J background). Then we determined the sizes and concentrations of the HDL particles by calibrated ion mobility (**Fig. 5A**).

The mean size of medium HDL from the wild-type mice was 9.8 nm (Fig. 5B). In contrast, it was 9.4 nm for the *Apoa2*^−/−^ mice and 10.1 nm for the transgenic mice expressing human APOA2. The mean sizes of the large HDLs exhibited the same trends, but the absolute differences were even more marked (Fig. 5C). These observations confirm that APOA2 has a key influence on the size of HDL. Plasma levels of both large and medium HDL were markedly reduced in the *Apoa2*^−/−^ mice (Fig. 5), as previously reported for HDL-C levels in these animals (37).

### The cholesterol efflux capacity of HDL varies significantly in different strains of mice

We used cAMP-stimulated J774 macrophages to quantify macrophage cholesterol efflux capacity of serum HDLs of the five strains of mice because this assay predicts both prevalent and incident human coronary artery disease (11, 14, 38). The differences were highly significant (Fig. 3B; ANOVA, F(4, 31) = 21.6, *P* < 0.0001). Serum HDL of C57BL/6J displayed the highest macrophage efflux capacity (6.2 ± 0.8%), while C57BLKS/J showed the lowest (4.7 ± 0.4%). The difference in efflux capacity between the two strains is similar to that observed between control subjects and subjects with incident or prevalent CVD (11, 13). Dunn’s post hoc analysis indicated significant differences in macrophage cholesterol efflux capacity between C57BL/6J and the other four strains (*P* < 0.0001), as well as between C57BLKS/J and SWR/J (*P* = 0.01).

Because the cholesterol efflux capacity of serum HDL with J774 macrophages reflects the activity of multiple cellular pathways and because human ABCA1 deficiency causes cholesterol-loaded macrophages to accumulate in many different tissues, we used BHK cells with mifepristone-inducible expression of human ABCA1 to quantify that pathway’s efflux capacity (Fig. 3C). In this system, ABCA1-specific cholesterol efflux capacity is calculated as the percentage of total [^3^H]cholesterol (medium plus cell) released into the medium of BHK cells stimulated with mifepristone minus the value obtained with cells incubated with medium. The differences in ABCA1-specific cholesterol efflux capacity between the strains of mice were highly significant [ANOVA, F(4, 31) = 7.9, *P* = 0.0002]. Serum HDL from DBA/2J mice had the largest efflux capacity (5.7 ± 0.5%), while serum HDL from NZW/LacJ mice had the lowest (3.7 ± 0.6%). Importantly, the rank orders of efflux capacity of serum HDLs with macrophages and ABCA1-expressing cells were also clearly different, strongly suggesting that different factors control HDL’s cholesterol efflux capacity of ABCA1-expressing BHK cells and J774 macrophages loaded with radiolabeled cholesterol.

We next examined the relationships between macrophage cholesterol efflux capacity, ABCA1-specific cholesterol efflux capacity, and macrophage ABCA1-specific cholesterol efflux capacity using serum HDL from all five strains of mice (14 animals). Macrophage cholesterol efflux capacity (efflux from J774 cells stimulated with cAMP) did not correlate (*r* = −0.09, *P* = 0.73) with ABCA1-specific cholesterol efflux capacity (efflux in mifepristone-stimulated BHK cells minus nonstimulated efflux). In contrast, ABCA1-specific cholesterol efflux of macrophages (efflux in cAMP-stimulated J774 macrophages minus nonstimulated efflux) correlated strongly (*r* = 0.86, *P* < 0.0001) with that of BHK cells. These observations confirm that cholesterol efflux from cAMP-stimulated J774 macrophages measures efflux by pathways distinct from ABCA1 (10). They also are in good agreement with our data indicating that different factors control macrophage and ABCA1-specific cholesterol efflux capacity (Fig. 3).

### HDL-C and APOA1 play different roles when they promote cholesterol efflux from macrophages or cells expressing high levels of ABCA1

Because the five strains of mice had significantly different levels of HDL-C, APOA1, and phospholipids in serum HDL (supplementary Fig. 2), we next investigated the relationship between these metrics, macrophage cholesterol efflux capacity, and ABCA1-specific cholesterol efflux capacity (supplementary Fig. 3). Macrophage cholesterol efflux with serum HDL correlated strongly with plasma HDL-C (Pearson’s correlation coefficient, *r* = 0.77, *P* < 0.0001) and phospholipids (*r* = 0.73, *P* < 0.0001), but not with plasma APOA1 (*r* = 0.41, *P* = 0.01) (supplementary Fig. 3A, C, E). In contrast, serum HDL’s ABCA1-specific cholesterol efflux capacity correlated strongly with APOA1 (*r* = 0.7, *P* < 0.001), but not with HDL-C (*r* = 0.2, *P* = 0.2) or phospholipids (supplementary Fig. 3B, D, F).

These observations support the proposal that ABCA1 accounts for only ∼30% of cholesterol efflux from J774 macrophages, as assessed with radiolabeled cholesterol (10). Importantly, 40–50% of the variance in serum HDL’s efflux capacity with macrophages or cells expressing ABCA1 is not predicted by HDL-C or APOA1 levels, strongly suggesting that other factors mediate cholesterol efflux in these systems.

### Identification of candidate HDL proteins that affect cholesterol efflux capacity

Because plasma APOA1, HDL’s major protein, failed to explain most of the variation in serum HDL’s macrophage efflux capacity, we next determined whether other HDL proteins might help modulate this HDL metric. Pearson’s analysis revealed that six HDL proteins detected by LC-ESI-MS/MS on the HDL isolated from the different strains of mice (Fig. 1) had significant (*P* ≤ 0.0001) and strong correlation coefficients (*r* ≥ |0.5|) with serum HDL’s efflux capacity with either macrophages or the ABCA1 pathway.

Two proteins, PLTP and RBP4, correlated strongly and positively (*r* = 0.49 and *r* = 0.60) with the cholesterol efflux capacity of serum HDL with macrophages (Fig. 4C, D), but showed lower correlations with efflux via the ABCA1 pathway (*r* = 0.44, *P* = 0.007 and *r* = 0.24, *P* = 0.15). In contrast, APOC3 (Fig. 4E) and APOD (Fig. 4F) in isolated HDL correlated strongly and positively (*r* = 0.78 and *r* = 0.62) with serum HDL’s efflux capacity with the cells that express ABCA1, but not with the macrophages (*r* = 0.2, *P* = 0.18 and *r* = 0.3, *P* = −0.07). Moreover, two proteins (Fig. 4G, H), APOE and SAA1, had strong negative correlations (*r* = −0.72 and *r* = −0.43) with efflux from the ABCA1-expressing cells, but showed little correlation with efflux from macrophages (*r* = −0.32, *P* = 0.05 and *r* = −0.204, *P* = 0.23). For example, DBA/2J mice exhibited the lowest HDL-associated APOE levels while having the largest (5.7 ± 0.5%, Fig. 3C) ABCA1-mediated cholesterol efflux capacity. These observations further support the proposal that HDL proteins play distinct roles in promoting cholesterol efflux from macrophages and cells expressing ABCA1.

### APOE modulates the efflux capacity of HDL with the ABCA1 pathway

**Fig. 6.**
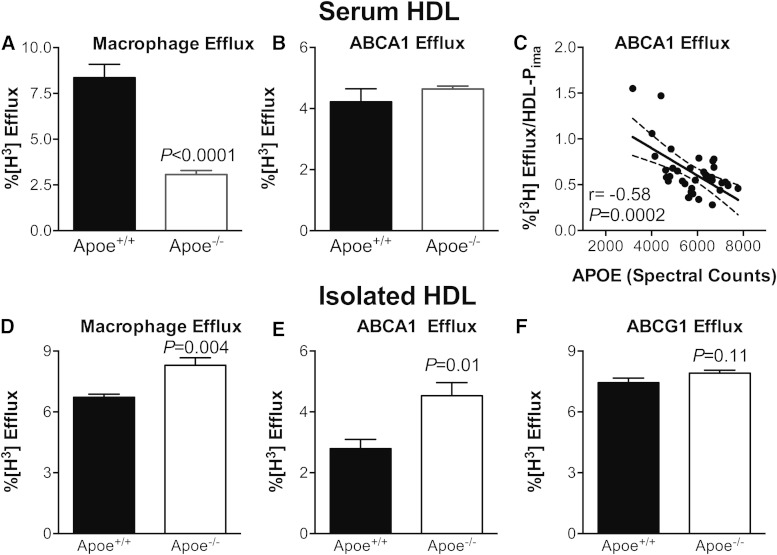
Macrophage and ABCA1-specific cholesterol efflux capacity of serum HDL (upper panels) and isolated HDL (lower panels). Macrophage cholesterol efflux capacity (A) and ABCA1-specific cholesterol efflux capacity (B) of serum HDL from wild-type and *Apoe*^−/−^ mice were measured as described in the legend to Fig. 3. C: Efflux capacity versus the APOE content of HDL isolated by ultracentrifugation from plasma. ABCA1-specific efflux capacity of HDL isolated from the different strains of mice is normalized to HDL particle concentration (HDL-P_ima_). D–F: Macrophage cholesterol efflux capacity, ABCA1-specific cholesterol efflux capacity, and ABCG1-specific cholesterol efflux capacity of HDL isolated by ultracentrifugation from the plasma of *Apoe*^+/+^ and *Apoe*^−/−^ mice. Cells were incubated with 30 μg HDL protein per milliliter.

Because APOE correlated negatively and strongly with ABCA1-specific efflux capacity of serum HDL (Fig. 4G), we quantified the efflux capacity of serum HDLs prepared from wild-type (*Apoe*^+/+^) and APOE-deficient (*Apoe*^−/−^) mice in the C57BL/6J background. The macrophage cholesterol efflux of serum HDL from the *Apoe*^−/−^ mice was markedly lower than that of wild-type mice (**Fig. 6A**). This difference likely reflects a decrease in the number of HDL particles, because HDL-C levels are markedly lower in *Apoe*^−/−^ than *Apoe*^+/+^ mice (39). When we normalized efflux capacity to HDL particle concentration (HDL-P_ima_ as determined by calibrated IMA), the efflux capacity of the HDL from the *Apoe*^−/−^ mice was higher than that of the HDL from the wild-type mice (9.68 ± 2.57% vs. 2.12 ± 1.02%, respectively, n = 3, *P* = 0.052), strongly suggesting that APOE expression impairs HDL’s efflux capacity with macrophages.

In striking contrast to efflux capacity with macrophages, the ABCA1-specific cholesterol efflux capacity of serum HDLs from *Apoe*^−/−^ and *Apoe*^+/+^ mice were similar (Fig. 6B), despite the markedly lower HDL-C and HDL particle concentration in these mice. When we used calibrated IMA to quantify HDL particle concentration in the *Apoe^+/+^* and *Apoe*^−/−^ mice, ABCA1-specific cholesterol efflux per particle correlated negatively and significantly (*r* = −0.58, *P* = 0.0002) with the APOE content of the HDL particles (Fig. 6C). When we normalized efflux capacity to HDL particle number in serum, ABCA1-specific cholesterol efflux was markedly higher in the *Apoe*^−/−^ mice than in the wild-type mice [9.71 ± 2.56 (n = 3) vs.1.56 ± 0.62 (n = 6), *P* = 0.038].

To determine the impact of APOE deficiency on the efflux capacity of HDL itself, we isolated HDL by ultracentrifugation from the plasma of *Apoe*^+/+^ and *Apoe*^−/−^ mice and measured macrophage efflux, ABCA1-specific efflux, and ABCG1-specific efflux, using cells exposed to identical protein concentrations of the lipoprotein (30 μg/ml). HDL isolated from the *Apoe*^−/−^ mice was 30% (*P *= 0.004) more efficient at promoting macrophage cholesterol efflux capacity than wild-type HDL (Fig. 6D). HDL isolated from the *Apoe*^−/−^ mice promoted ABCA1-specific efflux even more effectively (Fig. 6E). In contrast, HDL isolated from *Apoe*^−/−^ and *Apoe*^+/+^ mice exhibited similar activities with cells expressing ABCG1 (Fig. 6F). Taken together, these observations demonstrate that in mice, HDL associated APOE impairs the efflux capacity of HDL with macrophages by a mechanism selectively involving the ABCA1 pathway.

## DISCUSSION

We used five inbred mouse strains to investigate the relationships between HDL’s size, protein cargo, and cholesterol efflux capacity. Proteomic analysis of isolated HDL identified more than 70 proteins linked to lipid metabolism, the acute-phase response, proteinase inhibition, and the immune system, as originally described for human HDL (15). Using size-exclusion and phospholipid affinity chromatography, Gordon et al. (36) obtained similar findings for HDL isolated from C57BL/6J mice. These observations indicate that the HDLs of the two distantly related species carry the same functional categories of proteins. However, only 42 of the 72 proteins we detected in mouse HDL have been identified in human HDL (36), suggesting both qualitative and quantitative differences.

Hierarchical cluster analysis of the HDL proteome recapitulated the genealogy of the five mouse strains. We found significant variability in HDL size and the cholesterol efflux capacity of serum HDL, supporting the idea that these proposed metrics of HDL’s cardioprotective effects are under genetic control. Moreover, the lipoprotein’s content of specific proteins strongly correlated with each metric, raising the possibility that HDL’s protein cargo helps regulate its size and function. For example, the APOA2 content of HDL strongly correlated with HDL size, as previously observed (37, 40–43).

Using mice that were deficient in APOA2 or expressed human APOA2, we confirmed that the protein controlled the size of both the medium and large HDL subspecies detected in mice. Because mouse and human APOA2 are only ∼60% identical in sequence and APOA2 is dimerized in human, but not mouse, HDL, the impact of APOA2 on size appears to be independent of its precise sequence. The fact that HDL’s lipid composition varied little between the mouse strains strengthens the proposal that APOA2 is an important structural protein that regulates HDL’s size.

The major protein of HDL is APOA1, but its content did not correlate with serum HDL’s ability to remove cholesterol from macrophages. However, we noted a strong correlation between cholesterol efflux capacity and isolated HDL’s content of RBP4 and PLTP. In contrast, serum HDL’s ability to remove cholesterol from BHK cells that expressed ABCA1 correlated strongly with isolated HDL’s content of APOA1, APOC3, and APOD. These observations imply that the proteins that control HDL’s efflux capacity with macrophages differ from those that influence cholesterol removal via the ABCA1 pathway. This is consistent with the finding that the ABCA1 pathway accounts for only about one-third of serum HDL’s efflux capacity with J774 macrophages (10). Although it is generally believed that lipid-free or lipid-poor apolipoproteins initially interact with ABCA1, a recent report shows that small HDLs (HDL3b and HDL3c) are effective in removing cholesterol from ABCA1-expressing cells (7, 44).Thus, HDL size is also likely to be an important determinant of ABCA1-dependent cholesterol efflux.

Two proteins in isolated HDL, APOE, and SAA1 strongly and negatively correlated with cholesterol efflux capacity when we used serum HDL to accept cholesterol from cells selectively expressing ABCA1. There was no such correlation with SAA2. It is noteworthy that elevated blood levels of total SAA (proteins carried exclusively by HDL in humans) associate with increased CVD risk (45). We also found that the cholesterol efflux capacity of serum HDL from *Apoe*^−/−^ mice was comparable to that of wild-type mice with the ABCA1 pathway when we used the same concentration of serum HDL in the assays. Because of the marked differences in HDL-C and HDL particle concentration in the two strains (>50% reduction of both metrics in *Apoe*^−/−^ mice), these observations are consistent with the idea that APOE associates with HDL directly or indirectly inhibits HDL’s efflux capacity by the ABCA1 pathway.

The J774 macrophages we used to quantify cholesterol efflux capacity do not secrete APOE. Thus, cell-derived APOE would not affect the efflux capacity of serum HDL in our studies. This is important because mouse and human macrophages secrete APOE (46, 47) and lipid-free APOE (48, 49), like other lipid-free apolipoproteins, promotes cholesterol efflux by ABCA1 due to its α helical structure (6). Cell autonomous production of APOE promotes cholesterol efflux from hematopoietic stem cells (50), demonstrating the potential impact of macrophage-derived APOE on efflux capacity.

We used wild-type and *Apoe*^−/−^ mice to determine whether HDL-associated APOE helps regulate the cholesterol efflux capacity of serum HDL. HDL isolated by ultracentrifugation from *Apoe*^−/−^ mice had significantly better efflux capacity with macrophages (based on HDL’s protein content or HDL particle concentration) than did wild-type HDL. Moreover, its efflux capacity increased markedly with cells expressing ABCA1, but not with cells expressing ABCG1. These observations provide strong evidence that APOE associated with APOE impairs HDL’s cholesterol efflux capacity from macrophages by a pathway involving ABCA1. Further experiments are required to understand how APOE affects the cholesterol efflux capacity of HDL subspecies.

We previously demonstrated that HDL’s APOE content was elevated in HDL isolated from humans with established CVD (15, 21). Moreover, in a prospective study of CVD risk in subjects with established atherosclerosis, the APOE content of APOA1-containing HDL particles was a much stronger predictor of future cardiac events than HDL-C or LDL-cholesterol levels (51). These observations raise the possibility that APOE impairs HDL’s cardioprotective effects, perhaps in part by affecting cholesterol efflux capacity.

Significantly, HDL-associated APOE interacts very differently with the ABCG1 pathway (52, 53), where it acts in concert with LCAT to drive net cholesterol efflux by promoting cholesteryl esterification and an increase in HDL size. It remains to be determined whether the cholesterol efflux capacity of serum HDL with the ABCG1 pathway associates with cardiovascular risk.

HDL’s content of PLTP correlated strongly with its ability to promote cholesterol efflux from macrophages. This observation is consistent with the finding that PLTP facilitates ABCA1-dependent cellular cholesterol efflux in concert with HDL (54). In vitro, PLTP remodels medium-sized HDL particles, generating larger HDL particles and preβ HDL, a ligand for ABCA1 (55). If this reaction takes place in vivo, it could promote cholesterol efflux from macrophages.

We also found a strong correlation of RBP4 with serum HDL’s cholesterol efflux capacity when we used macrophages. RBP4 contributes to the insulin resistance that develops in adipose-specific *Glut4*^−/−^ mice, but little is known about its role in cholesterol transport and HDL function (56)

Our observations demonstrate the power of proteomics in concert with genetics to identify proteins that affect HDL’s structure and function. These findings may be clinically relevant, because HDL’s APOE content is a strong predictor of CVD risk and because ABCA1 deficiency promotes unregulated cholesterol accumulation in human macrophages. In future studies, it will be of interest to determine whether HDL proteins promote cholesterol efflux from macrophages in vivo and to establish whether variations in HDL’s proteome could identify subjects at increased risk of CVD better than HDL-C, the current clinical gold standard.

## Supplementary Material

Supplemental Data
