# Supplementary material for: Proteomic analysis of HDL from inbred mouse strains implicates APOE associated with HDL in reduced cholesterol efflux capacity via the ABCA1 pathway
Source: J Lipid Res. 2016 Feb;57(2):246–57. doi: 10.1194/jlr.M063701 (PMC4727420; doi:10.1194/jlr.M063701)
Supplement: Supplemental Data [file supp_57_2_246__index.html]

Proteomic analysis of HDL from inbred mice strains implicates APOE associated with HDL in reduced cholesterol efflux capacity via the ABCA1 pathway — Proteomic analysis of HDL from inbred mouse strains implicates APOE associated with HDL in reduced cholesterol efflux capacity via the ABCA1 pathway — Supplemental Data 

# Proteomic analysis of HDL from inbred mouse strains implicates APOE associated with HDL in reduced cholesterol efflux capacity via the ABCA1 pathway

## Supplemental Data

- Supplemental table (.xlsx, 8.7 MB) - Supplemental table containing the mass spectrometry data
- supplemental figures (.pdf, 360 KB) - supplemental figures
