# Supplementary material for: Proteomic analysis of HDL from inbred mouse strains implicates APOE associated with HDL in reduced cholesterol efflux capacity via the ABCA1 pathway
Source: J Lipid Res. 2016 Feb;57(2):246–57. doi: 10.1194/jlr.M063701 (PMC4727420; doi:10.1194/jlr.M063701)

Supplemental Table and Figure Legends

Supplemental Table 1. Proteins detected by LC-ESI MS/MS in the 5 strains of inbred mice.

All protein identifications required: (i) detection of at least 2 unique peptides for each protein from at least three different mice and (ii) identification in $\geq$75% of mice in at least one strain of animals. Spectral counts for each protein are normalized to total peptide spectral counts in each analysis and represent relative abundance. Data are mean±SD. Significance was assessed by non-parametric ANOVA and the Kruskal Wallis test.

Supplemental Figure 1. Cluster analysis of the relative levels of proteins in HDLs in the five strains of inbred mice. The relationships were determined by Euclidean distance clustering.

Supplemental Figure 2. HDL lipid profiles of the 5 strains of inbred mouse strains. (**A-E**) Lipids and APOA1 were measured in serum depleted of APOB containing lipoproteins. (**F**) Percent of lipid moieties and protein in ultracentrifugation isolated HDL.

Supplemental Figure 3. Correlations of serum HDL-C, APOA1 and phospholipids with macrophage and ABCA1-specific cholesterol efflux capacities. Serum HDL was obtained by polyethylene glycol (PEG) precipitation of APOB containing lipoproteins from plasma-derived serum. Efflux of [^{3}H]cholesterol was measured after a 4 h incubation in medium with serum HDL (2.8% v/v) as described in Methods. The relationship of each HDL metric to efflux capacity was quantified by Pearson's correlation.

Supplemental Figure 1

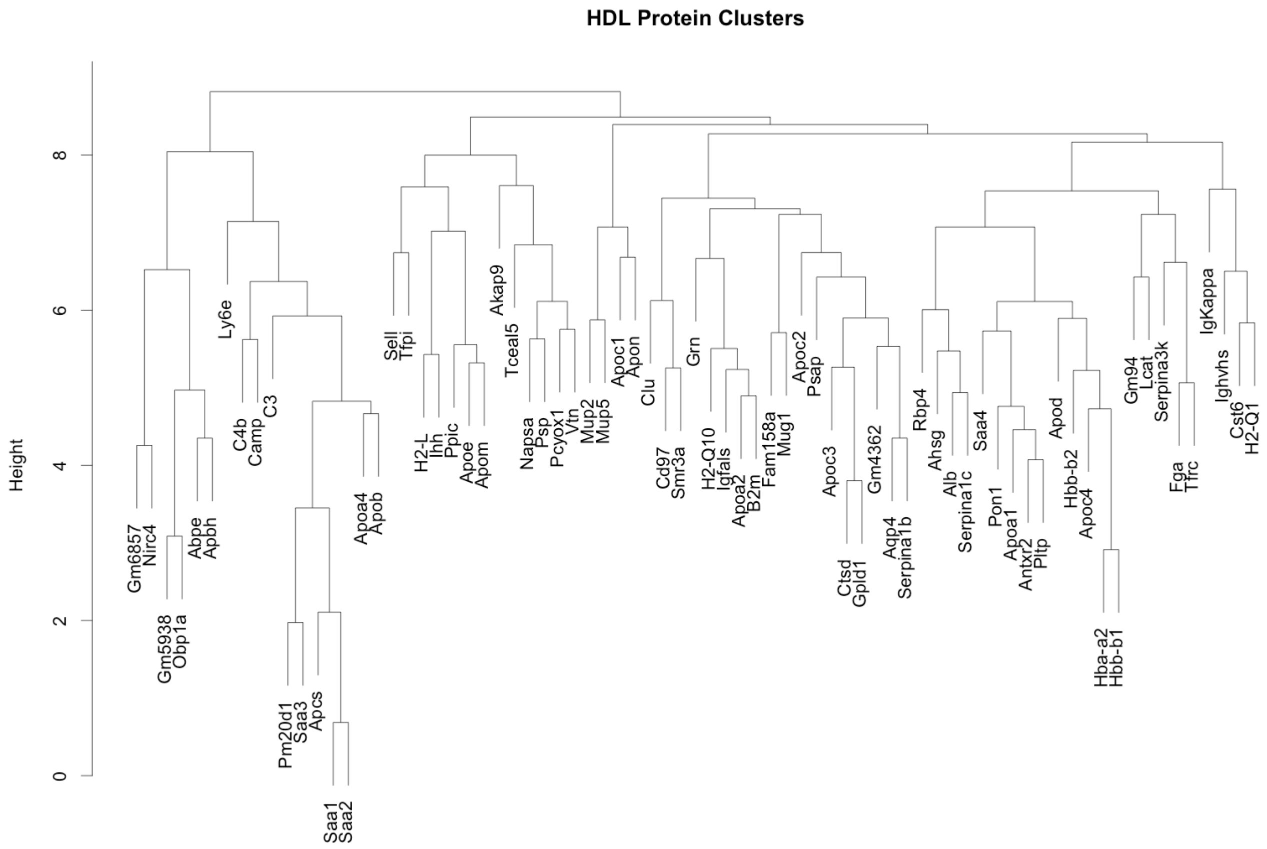

Supplemental Figure 2

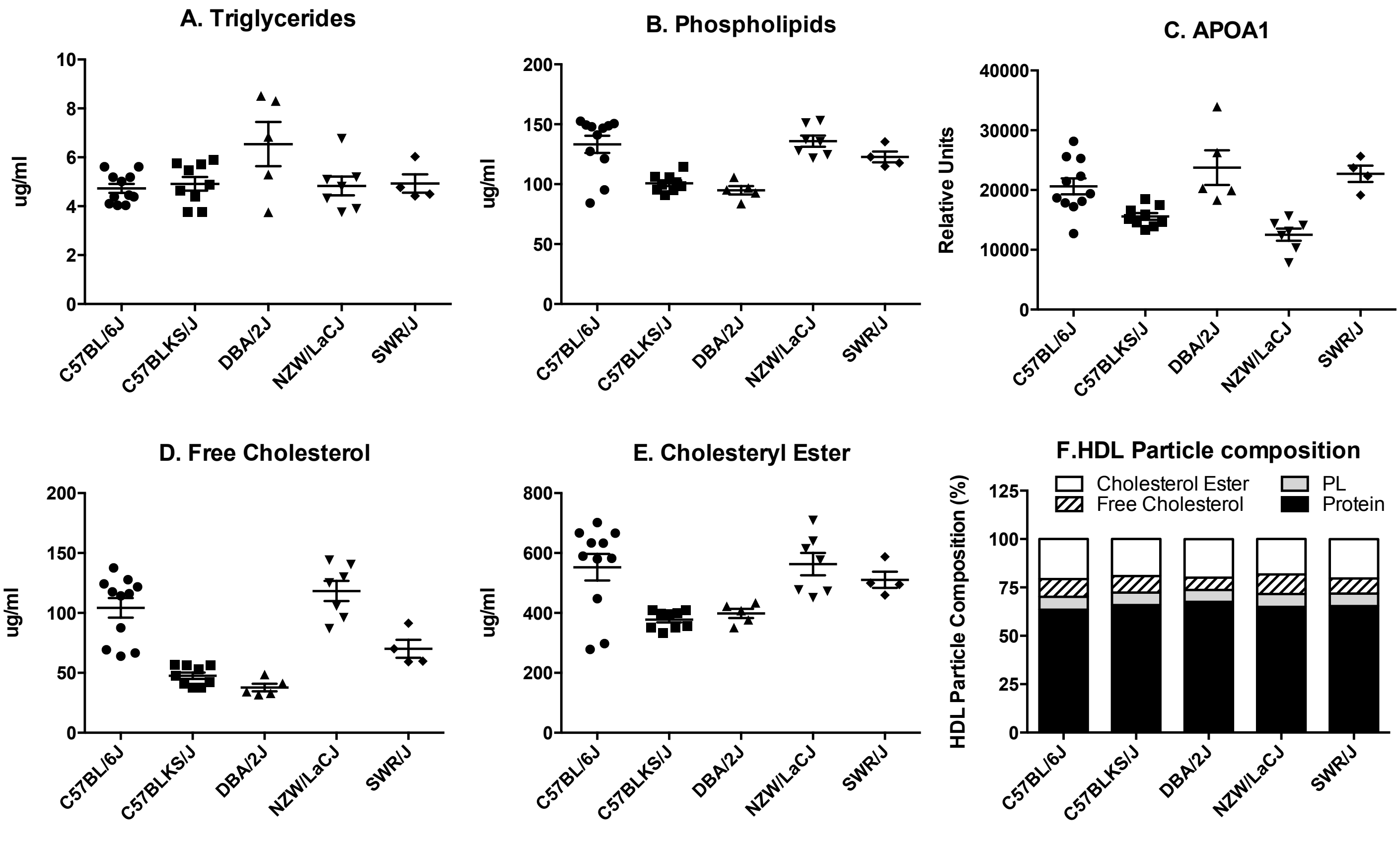

Supplemental Figure 3

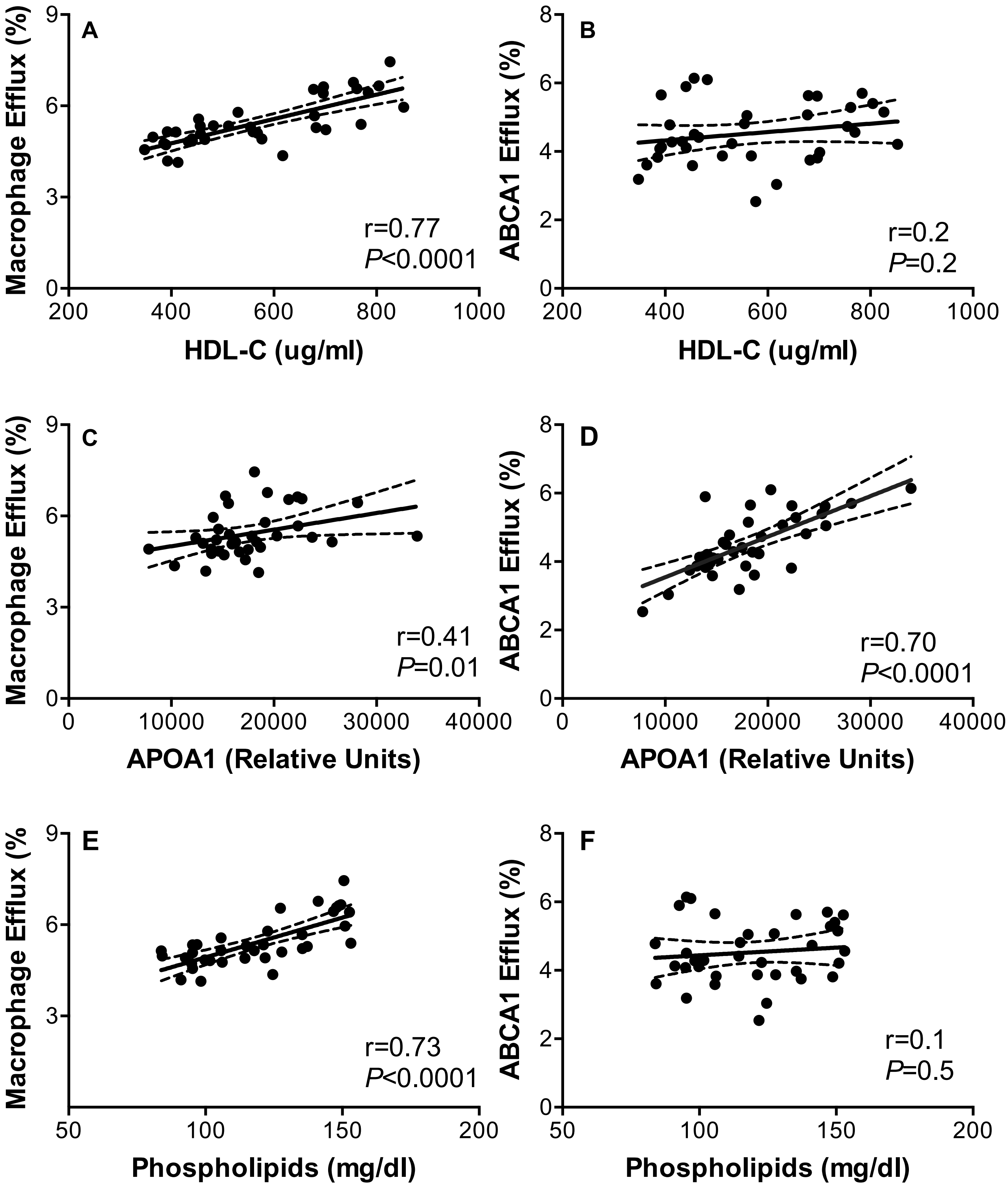

Supplement: Supplemental Data [file 10.1194_M063701_jlr.M063701-2.pdf]
